# Macrophages in Health and Non-Infectious Disease 2.0

**DOI:** 10.3390/biomedicines10061215

**Published:** 2022-05-24

**Authors:** Evgeny E. Bezsonov, Alexei Gratchev, Alexander N. Orekhov

**Affiliations:** 1 Laboratory of Angiopathology, Institute of General Pathology and Pathophysiology, 8 Baltiiskaya Street, 125315 Moscow, Russia; 2 Federal State Budgetary Scientific Institution “Petrovsky National Research Centre of Surgery”, 3 Tsyurupa Street, 117418 Moscow, Russia; 3 Department of Biology and General Genetics, I. M. Sechenov First Moscow State Medical University (Sechenov University), 8 Izmailovsky Boulevard, 105043 Moscow, Russia; 4 N.N. Blokhin Cancer Research Center, Institute of Carcinogenesis, 115478 Moscow, Russia; alexei.gratchev@gmail.com

This Special Issue (SI) has collected the most recent publications on the mechanisms that macrophages use to regulate homeostasis and their involvement in the pathogenesis of various non-infectious diseases.

Among others, atherosclerosis can be considered as an inevitable disease, strongly associated with age, and significantly contributing to the death rate of all countries.

The pathological thickening of the walls of large arteries can be seen as a macroscopic manifestation of this disease, and when we increase the scale we can see that macrophages contribute significantly by secreting pro-inflammatory cytokines leading to chronic inflammation, participating in the formation of atherosclerotic lesions in the form of foam cells [1,2]. However, when it comes to the identification of the origins of atherosclerosis, there is still no unity among researchers.

One promising hypothesis combining different observations related to atherosclerosis pathology is that mutations of mitochondrial DNA could modulate the inflammatory response of macrophages to different pro-inflammatory stimuli, leading to chronic inflammation and further development of the disease [3,4]. As a part of the innate immune system, macrophages are capable of recognizing pathogen-associated molecular patterns (PAMPs) and damage-associated molecular patterns (DAMPs), and can trigger immune response. Dysfunctional mitochondria (for example, due to mutations of mitochondrial DNA) contribute to DAMPs formation. Atherogenic low-density lipoproteins (LDL), including desialylated and oxidized ones [2,5], can also serve as a unique trigger of inflammation independent of DAMPs and PAMPs. It should be noted that the principles described in this hypothesis can also be applied to other diseases related to chronic inflammation. This hypothesis is shown in Figure 1.

We shall briefly summarize below articles and reviews published in this Special Issue.

It was found that infection of anti-inflammatory M2-macrophages with rubella virus was accompanied by a reduction in CD14 expression and the interferon β response [6].

Specific gene profiles were identified in heart macrophages in cases of early compensated hypertrophy (genes related to lipid metabolism and genes of Na^+^ or K^+^ channels) versus late dilated remodeling related to heart failure [7].

The influence of heat-killed *Candida albicans* cells on macrophages was studied with the conclusion that these cells can induce foam cell formation, MMP-9 expression, and inflammatory response via upregulated FABP4. Thus, FABP4 could be considered as a new drug target to treat atherosclerosis induced by *C. albicans* [8].

The potential connection of high levels of lipid peroxidation with elevated ferritin was investigated in case of adult-onset Still’s disease [9].

The influence of lobeglitazone on lipopolysaccharide-treated bone-marrow-derived macrophages was studied, with the conclusion that it has anti-inflammatory activity due to the suppression of expression of pro-inflammatory genes and reduced NO production [10].

Mice-originating tissue-resident macrophages specific for lungs were co-incubated with lung interstitial cells in order to study the microenvironment of the expression of specific markers for cell lines studied [11].

The expression of the MARCO gene (as well as some other markers) in macrophages originating from liver after a resection procedure was investigated, with a conclusion regarding the increase in MARCO in these cells upon regeneration of liver in a mouse model [12].

Using a mouse model, a line of human macrophages, and an agonist of the glucose-dependent insulinotropic polypeptide (GIP) receptor, a reduction in foam cell formation was shown upon the activation of the GIP receptor, which correlated with a reduction in expression of *CD36* and *CDK5* [13].

Macrophages were purified from different regions of lungs based on density of cells, with the finding that high-density and low-density macrophages had differences not only in density but also in the expression of certain gene markers and inflammatory response upon the addition of lipopolysaccharide [14].

Co-incubation of mesenchymal stem cells with M2 macrophages in a 3D environment containing polyethylene particles with lipopolysaccharides resulted in an elevated production of markers of osteogenesis. This suggests a potential role of the immune response in modulation of bone reparation [15].

It was found that incubation of macrophages with carnosine led to a reduction in oxidative stress caused by Aβ1-42 oligomers and thus protected cells from death and apoptosis. This points in the direction of potential therapeutic application of this peptide in the treatment of Alzheimer’s disease [16].

There was increased production of interleukin-8 and prostaglandin E_2_ in THP-1-derived macrophages after palmitate treatment, and insulin enhanced these effects, suggesting the role of palmitate in the progression of inflammation in adipose tissues [17].

The pathological role of macrophages in development and progression of erectile dysfunction (ED), and Peyronie’s disease as an example of ED, was reviewed and discussed, including current therapeutic approaches [18].

The complex subject of training and tolerance of macrophages was discussed with special focus on the role of macrophages in the development of diseases in humans [19].

Questions related to metabolism of iron in macrophages, considering their role in degradation of red blood cells, were carefully reviewed [20].

The influence of activation of microglia on pathological development of neurodegenerative diseases such as Parkinson’s disease, Alzheimer’s disease, Huntington’s disease and epilepsy was discussed, with mention of potential therapy approaches [21].

Current methods related to the identification of macrophages in situ were systematized and discussed, including in situ hybridization, immunolabeling and other approaches [22].

The interaction between macrophages, mesenchymal stem cells and fibroblasts in the tumor microenvironment was analyzed, with a focus on cell–cell interaction and secreted mediators, in order to find explanations for pro/anti-tumor phenotypes of macrophages and their response to treatment with oncolytic viruses [23].

The role of macrophages in foam cell formation in atherosclerosis, mechanisms involved in this process, as well as their potential as targets for anti-atherosclerotic therapy, were reviewed [24].

The effect of Roux-en-Y gastric bypass on different pathways involving energy homeostasis in leukocytes was studied, with the finding of increased levels of AMPK, autophagy/mitophagy markers, a reduction in ATF6 and CHOP (ER stress markers), and decreased mitochondrial membrane potential [25].

The influence of microbeam radiation therapy on accumulation of macrophages was studied in different tissues, and accumulation of macrophages in normal liver and lung tissue and lung carcinoma was found in comparison to in normal skin tissue [26].

We hope that the next SI will continue the traditions of high-quality publications established in this and previous SIs.

## Figures and Tables

**Figure 1:**
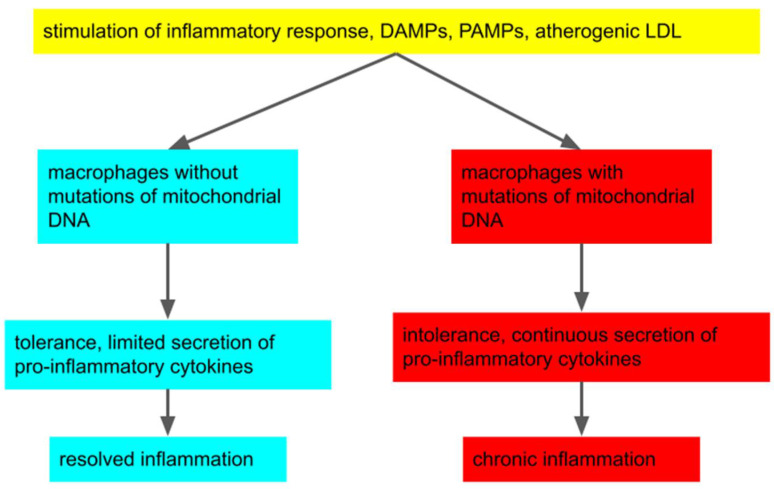
The hypothesis of the induction of chronic inflammation due to the presence of mutations of mitochondrial DNA in macrophages. Macrophages can recognize damage-associated molecular patterns (DAMPs), pathogen-associated molecular patterns (PAMPs), and atherogenic low-density lipoproteins (LDL), turn on a pro-inflammatory reaction, and secrete pro-inflammatory cytokines. In normal cases, a tolerance develops, and the macrophage stops releasing cytokines. However, in the presence of mitochondrial mutations, tolerance formation may be impaired, which could lead to continuous secretion of inflammatory cytokines and chronic inflammation development [3,4].
